# CMR feature tracking in cardiac asymptomatic systemic sclerosis: Clinical implications

**DOI:** 10.1371/journal.pone.0221021

**Published:** 2019-08-21

**Authors:** Konstantinos Bratis, Anthony Lindholm, Roger Hesselstrand, Håkan Arheden, Georgia Karabela, Efthymios Stavropoulos, Gikas Katsifis, Genovefa Kolovou, George D. Kitas, Petros P. Sfikakis, Loukia Koutsogeorgopoulou, Sophie Mavrogeni, Ellen Ostenfeld

**Affiliations:** 1 CMR Unit, Onassis Cardiac Surgery Centre, Athens, Greece; 2 Department of Clinical Sciences, Clinical Physiology, Skåne University Hospital, Lund University, Lund, Sweden; 3 Department of Clinical Sciences, Rheumatology, Skåne University Hospital, Lund University, Lund, Sweden; 4 Naval Hospital, Athens, Greece; 5 Arthritis Research UK Epidemiology Unit, University of Manchester, Manchester, United Kingdom; 6 First Department of Propeudeutic and Internal Medicine, Laikon Hospital, Athens University Medical School, Athens, Greece; 7 Pathophysiology Department, Laikon Hospital, Athens, Greece; University of Palermo, ITALY

## Abstract

**Background:**

Impaired myocardial deformation has been sporadically described in cardiac asymptomatic systemic sclerosis (SSc). We aimed to study myocardial deformation indices in cardiac asymptomatic SSc patients using cardiac magnetic resonance feature tracking (CMR-FT) and correlate these findings to the phenotypic and autoimmune background.

**Methods:**

Fifty-four cardiac asymptomatic SSc patients (44 females, 56±13 years), with normal routine cardiac assessment and CMR evaluation, including cine and late gadolinium enhancement (LGE) images, were included. SSc patients were compared to 21 sex- and age- matched healthy controls (17 females; 54±19 years). For CMR-FT analysis, a mid-ventricular slice for LV peak systolic radial and circumferential strain and a 4-chamber view for LV/RV peak systolic longitudinal strain were used.

**Results:**

Twenty-four patients had diffuse cutaneous SSc and 30 limited cutaneous SSc. Thirteen patients had digital ulcers. Median disease duration was 3.6 years. LV ejection fraction was higher in SSc patients compared to controls (62±6% vs. 59±5%, p = 0.01). Four patients had no LGE examination; in the remaining patients LGE was absent in 74%, while 18% had RV insertion fibrosis and 8% evidence of subendocardial infarction. LV longitudinal strain differed in those with insertion fibrosis (-18.0%) and infarction (-16.7%) compared to no fibrosis (-20.3%, p = 0.04). Patients with SSc had lower RV longitudinal strain and strain rate compared to controls (p<0.001 and p = 0.01, respectively). All other strain and strain rate measurements were non-significant between patients and controls.

**Conclusions:**

In cardiac asymptomatic SSc patients with normal routine functional indices, CMR-FT identifies subclinical presence of insertion fibrosis and/or myocardial infarction by impaired LV longitudinal strain. RV derived longitudinal indices were impaired in the patient group. CMR FT indices did not correlate to the patients’ phenotypic and autoimmune features.

## 1. Introduction

The heart is a major target organ in systemic sclerosis (SSc), appearing to be involved in 12–80% of autopsy studies [1], although the involvement is often clinically silent [2] and is recognized only in 15–25% [3, 4]. Myocardial disease is complex and dynamic and includes myositis, characterized by immune-mediated myopericardial inflammation [5, 6], microvascular dysfunction [7–10] and fibrosis [4] predisposing to cardiac dysfunction and failure, coronary artery disease, conduction system abnormalities and pericardial disease [11]. All subtypes of SSc are at risk for significant heart disease, but patients with rapidly evolving diffuse skin disease [12] as well as those with underlying skeletal muscle disease [13, 14] are prone to develop severe cardiomyopathy. Cardiac involvement carries an ominous prognosis, irrespective of the clinical presentation [15]. Early detection allows to timely start an immunosuppressive treatment and possibly prevent cardiac damage progression [16].

Cardiovascular magnetic resonance (CMR) is an accurate method for non-invasive, non-radiating assessment of ventricular volumes, function, myocardial perfusion as well as tissue characterization [17]. While ejection fraction is of prognostic value, it is a crude measure of subtle myocardial changes. Feature tracking derived from CMR cine images (CMR-FT) offers quantitative assessment of the myocardial deformation, beyond global assessment with ejection fraction and before other recognized markers [18, 19].

In the setting of SSc, there is only limited data [20] examining CMR derived left (LV) and right ventricular (RV) deformation indices in patients with SSc compared to healthy controls and their relationship to clinical subsets (diffuse cutaneous SSc (dcSSc) and limited cutaneous SSc (lcSSc)) and other disease features (presence of digital ulcers, disease duration, antibody subset). The aims of this study were 1) to investigate if LV longitudinal, radial and circumferential as well as RV longitudinal strain differ in cardiac asymptomatic SSc patients with preserved ejection fraction and normal estimated pulmonary pressure compared to healthy controls, 2) to correlate the LV and RV deformation indices to clinical subsets (lcSSc vs dcSSc) and other disease characteristics (digital ulcers, autoimmune profile, disease duration, unknown myocardial fibrosis) and assess its potential clinical value.

## 2. Materials and methods

### 2.1. Patients and controls

The study was conducted at Skåne University Hospital, Lund, Sweden and Onassis Cardiac Surgery Centre, Athens, Greece and patients were included from both hospitals. Patients fulfilling the American College of Rheumatology criteria [21] and/or LeRoy’s classification criteria for the diagnosis of SSc [22], who were prospectively included in prior studies from our groups [17, 23, 24] and had undergone a CMR exam were retrospectively included and analysed provided they had normal routine cardiac assessment and no cardiac symptoms. Exclusion criteria were known heart disease, renal failure, pulmonary hypertension and contraindications to CMR.

Medical records were reviewed to collect clinical characteristics of the patients. Detailed history, physical examination, routine laboratory investigations, autoimmune screening as well as screening for atherosclerotic disease risk factors were assessed in all patients. For subgroup analyses, patients were divided based on the observation time from SSc diagnosis to CMR examination (disease duration), skin involvement (lcSSc vs dcSSc), presence of digital ulcers and autoimmune profile [anti centromerantibodies (ACA), anti-nuclear antibodies (ANA), anti RNA polymerase III antibodies (ARA), anti-topoisomerase I antibodies (ATA))]. SSc patients were compared with sex-matched healthy controls that underwent cine CMR evaluation during the same period. Controls were prospectively enrolled in previous study from the Lund group [25] and were matched with the patient population for sex and age, as LV strain with CMR has been shown to be sex dependent [26]. Controls were checked for and had no cardiac morbidities, medical history or medication and were examined by clinicians before their study enrollment.

This study was approved by the ethics committees of Skåne University Hospital, Lund, Sweden and Onassis Cardiac Surgery Centre, Athens, Greece in accordance with the ethical guidelines of the 1975 Declaration of Helsinki. Informed written consent was obtained from patients and healthy controls.

### 2.2. CMR protocol

CMR was performed on a 1.5 Tesla scanner using ECG-triggered cine steady-state free precession breath-hold cine long-axis planes and sequential 8 mm short-axis slices including the atrioventricular ring to the apex to assess ventricular function. Typical image parameters were: echo time (TE) 1.4–1.6, repetition time (TR) 2.8–3.2 ms, flip angle 60° and gap 0 mm (Achieva, Philips Medical Healthcare, Best, the Netherlands), 2 mm (Aera, Siemens, Erlangen, Germany) and 3 mm (GE hdxt 1.5 T, version 16, GE Healthcare, Milwaukee, WI, USA).

To assess for fibrosis, late gadolinium enhanced (LGE) images were acquired 10–15 minutes after intravenous administration of gadolinium-DOTA (Dotarem, Guerbet, Roissy, France; 0.2mmol/kg) in identical short-axis planes using an inversion-recovery gradient echo sequence for fibrosis detection. Inversion times were adjusted to null normal myocardium.

### 2.3. CMR image analysis

**Fig 1:**
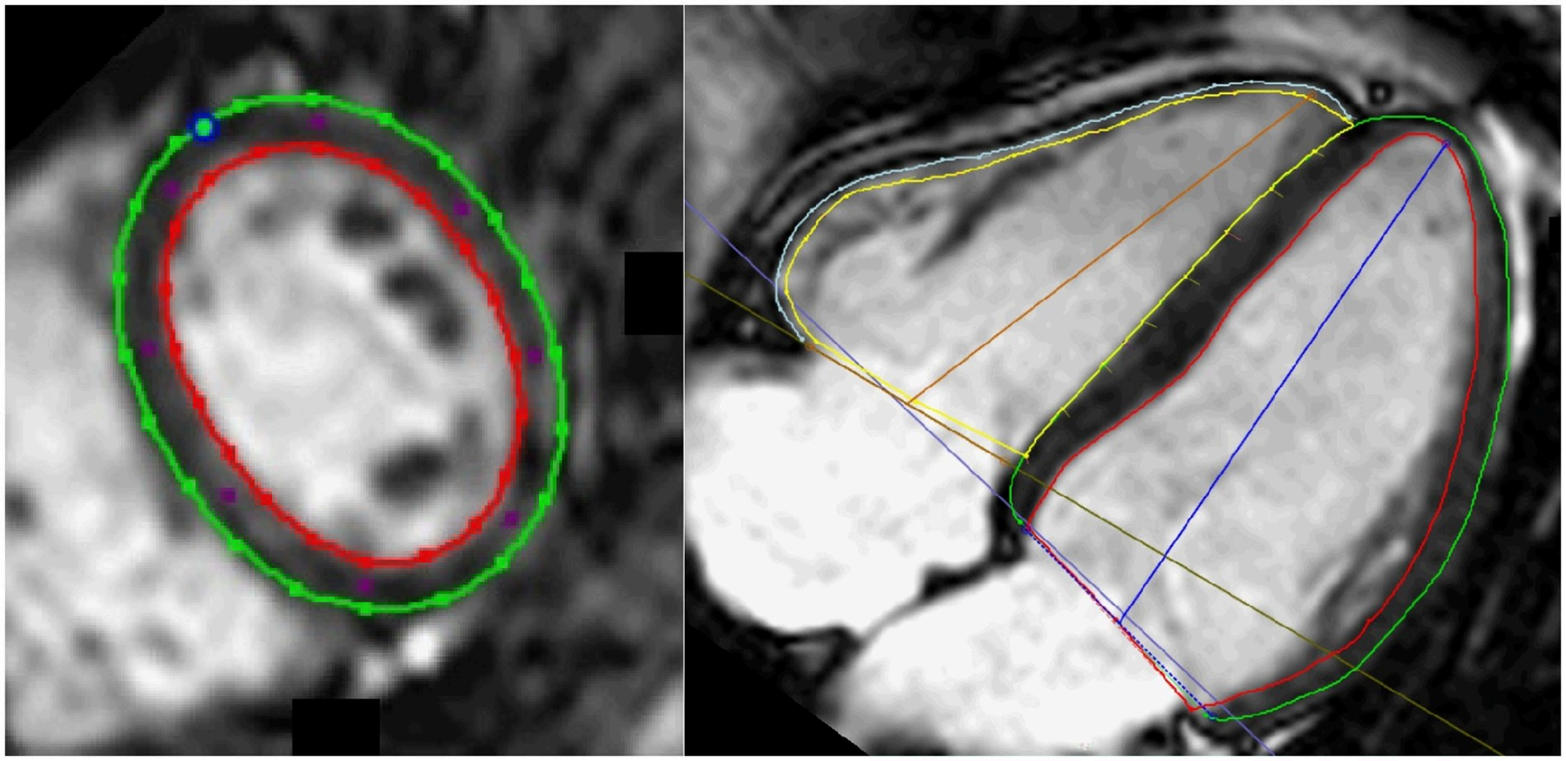
Example of delineations in one slice from a short axis stack (left) and 4-chamber long axis view (right). Green line represents left ventricle epicardial border, red line represents left ventricular endocardial border. Blue circle represents the insertion point in the short axis between the right and left ventricle and blue lines represent left ventricle atrioventricular plane and ventricular axis to the apex. Yellow line represents right ventricle endocardial border, light blue line represents right ventricle epicardial border and the brown line represents the right ventricle atrioventricular plane and ventricular axis to the apex.

CMR studies were analysed with Circle cmr^42^ 5.3.4 Tissue-Tracking Plugin (Circle Cardiovascular Imaging Inc., Calgary, AB, Canada) in a random order blinded to the patient clinical characteristics (A.L.). Cine short axis was used to evaluate LV and RV ejection fractions (EF). Analyses of myocardial deformation from two-dimensional strain and strain rate data were performed using feature-tracking imaging. Peak systolic LV and RV longitudinal myocardial deformation were measured in the left 4-chamber view (Fig 1). LV longitudinal deformation was the average of 7 segments, while the RV deformation was the average of RV free wall alone. Peak systolic LV radial and circumferential deformation indices were measured in LV short axis view at the mid ventricular level and were expressed as the average of six segments. The intrinsically negative measured data were converted to absolute values. In case of misalignment, an experienced observer acted as a blinded independent adjudicator (E.O.). For interobserver variability second observer (K.B.) blinded to prior data analyzed deformation indices on 15 individuals (10 patients and 5 controls). The evaluation of scar tissue was performed visually from the late gadolinium enhancement images (E.O.) and was categorized as absent LGE, fibrosis at RV insertion and subendocardial or transmural infarction.

### 2.4. Estimation of pulmonary pressure

Echocardiography was performed on clinical indication and/or as screening for pulmonary arterial hypertension. Pulmonary pressure was estimated from trans-tricuspid regurgitation with maximum velocity (TRVmax). TRVmax ≤ 2.8 m/s was considered normal estimated pulmonary pressure, while TRVmax > 2.8 m/s giving rise to suspected elevated pressure [27]. Patients with suspected elevated pressure had invasive right heart catheterisation performed by clinical indication. Mean pulmonary arterial pressure ≥25 mmHg was considered elevated [28] and patients with increased pulmonary pressure were not included.

### 2.5. Statistical analysis

All data are reported as mean ± SD. Statistical analysis was performed using SPSS software (SPSS Inc., Chicago, IL, USA, version 23.0). Correlation analysis was assessed using Pearson’s correlation. Volumetric measurements were normalized to the body surface area. Chi-square and unpaired student t tests were used to compare groups. ANOVA was used to test differences among groups. Mean ± SD was calculated to study the variability of the measurements. Inter-observer variability was assessed using intra class correlation coefficient. Results with a p-value of < 0.05 were considered statistically significant.

## 3. Results

### 3.1. Patient and controls

Fifty-four cardiac asymptomatic SSc patients (44 females, aged 56 ± 13 years) with no known cardiac involvement were retrospectively enrolled into the study. Twenty-one age- and gender- matched healthy controls were used for comparison [25]. Demographic characteristics are shown in Table 1. CMR functional characteristics of the included patients and control subjects are shown in Table 2.

**Table 1:** Patient characteristics.

	Controls (n = 21)	SSc (n = 54)	P-value
Sex (female)	17 (80%)	44 (81%)	1.0
Age (years)	54±19	56±13	0.8
BSA (m^2^)	1.8±0.2	1.8±0.2	0.7
Heart rate (bpm)	63±8	79±79	<0.001
***CMR***			
Fibrosis/infarction	n/a	9 (18%) / 4 (8%)	n/a
LV mass	79±19	84±27	0.4
***SSc Phenotype***			
SSc-duration (months)	n/a	43±70	n/a
mRSS	n/a	5±8	n/a
Raynaud’s	n/a	48 (89%)	n/a
SSc-type (diffuse/limited)	n/a	24 (44%) / 30 (56%)	n/a
Digital ulcer	n/a	13 (24%)	n/a
***Comorbidities***			
Smoker (yes/ex)	n/a	7 (13%) / 11 (20%)	n/a
Diabetes	n/a	9 (17%)	n/a
COPD/Emphysema	n/a	11 (20%)	n/a
Hypertension	n/a	8 (15%)	n/a
Known IHD	n/a	1 (2%)	n/a
***Medication***			
ACE/ARB	n/a	12(22%)	n/a
CCB	n/a	27(50%)	n/a
BB	n/a	2(4%)	n/a
Statin	n/a	11(20%)	n/a
NSAID	n/a	13(24%)	n/a
Corticosteroid	n/a	12(22%)	n/a
Immunosuppressant	n/a	11(20%)	n/a

Expressed as mean±SD or absolute numbers with percentage in parenthesis.

Control = Healthy adult volunteers; SSc = Systemic sclerosis; BSA = Body surface area; LV mass = Left ventricular mass; CMR = Cardiac magnetic resonance; mRSS = Modified Rodnan skin score; COPD = Chronic obstructive pulmonary disease; IHD = Ischemic heart disease; SSc-duration in years; ACE/ARB = Ace inhibitor/Angiotensin II blocker; CCB = Calcium channel blocker; BB = Beta-blocker; ERA = Endothelin-receptor antagonists; PDEI5 = Phosphodiesterase type 5 inhibitor; NSAID = Non-steroidal anti-inflammatory drugs, n/a = not applicable

**Table 2:** Cardiac magnetic resonance (CMR) functional, volumetric and myocardial deformation data in healthy adult volunteers (control), systemic sclerosis (SSc) patients and subgroups (diffuse and limited cutaneous systemic sclerosis (SSc) patients, SSc patients with and without digital ulcers).

	Controls (n = 21)	SSc (n = 54)	P-value^A^	Diffuse SSc (n = 24)	Limited SSc (n = 30)	P-value^B^	No Digital Ulcer (n = 41)	Digital Ulcer (n = 13)	P-value^C^
**Left ventricle**									
LVEF (%)	59±5	62±6	0.01	63±5	62±7	0.4	61±6	65±3	0.02
LVEDV (ml)	156±26	131±28	0.001	131±25	131±30	0.8	132±28	129±26	0.7
LVESV (ml)	65±14	50±15	< 0.001	49±12	51±16	0.7	51±15	46±11	0.2
LVSV (ml)	89±17	79±17	0.04	84±13	78±18	0.3	79±17	83±17	0.5
Strain (Mid radial)	44.5±8.5 (n = 19)^D^	42.2±11.8 (n = 52)^E^	0.4	38.7±12.2 (n = 22)	44.8±11.0	0.07	43.4±12	37.9±11 (n = 11)	0.2
Strain rate (Mid radial)	2.4±0.72 (n = 19)	2.5±0.82 (n = 47)^F^	0.7	2.3±0.75 (n = 17)	2.6±0.85	0.2	2.5±0.80 (n = 38)	2.3±0.91 (n = 9)	0.3
Strain (Mid circumferential)	-21.9±2.8 (n = 19)	-21.6±3.2 (n = 52)^E^	0.7	-20.9±3.7 (n = 22)	-22.1±3.0	0.2	22.1±3.0	-19.7±3.4 (n = 11)	0.02
Strain rate (Mid circumferential)	-1.0±0.57 (n = 19)	-1.3±0.37 (n = 47)^F^	0.07	-1.2±0.42 (n = 17)	-1.3±0.34	0.6	1.3±0.37 (n = 38)	-1.1±0.34 (n = 9)	0.1
Strain (Longitudinal)	-20.3±2.5	-19.4±3.2	0.3	-18.9±2.9	-19.8±3.4	0.3	19.7±3.1	-18.8±3.4	0.4
Strain rate (Longitudinal)	-1.1±0.21	-1.2±0.35 (n = 49)^G^	0.5	-1.1±0.36 (n = 19)	1.1±0.28	0.6	1.1±0.32 (n = 38)	-1.1±0.29 (n = 11)	0.5
**Right ventricle**									
RVEF (%)	58±6	59±9	0.8	56±10	60±7	0.08	59±7	57±12	0.4
RVEDV (ml)	164±34	129±37	< 0.001	131±49	127±25	0.7	133±39	115±31	0.1
RVESV (ml)	68±19	54±26	0.04	59±35	51±16	0.3	56±26	50±25	0.5
RVSV (ml)	94±23	74±17	< 0.001	72±20	76±15	0.4	77±16	65±18	0.03
Strain (Longitudinal)	-28.2±2.0 (n = 20)^H^	-27.0±4.0	0.01	-25.9±4.4	-27.9±3.4	0.07	27.4±3.9	-26.0±4.2	0.3

Strain is measured in (%), Strain rate is measured in (1/s) expressed in mean±SD.

^A^: controls versus all SSc,

^B^: diffuse versus limited cutaneous SSc,

^C^: without versus with digital ulcers.

^D^: Two controls had inadequate image acquisition for radial and circumferential tracking,

^E^: Two patients had artefacts,

^F^: 7 had no time data for strain rate analysis in short axis images,

^G^: Five patients had not time data for strain rate analysis in 4-chamber view,

^H^: One control had inadequate tracking for RV strain analysis.

Control = Healthy adult volunteers; SSc = Systemic sclerosis; LVEF = Left ventricular ejection fraction; LVEDV = Left ventricular end diastolic volume; LVESV = Left ventricular end systolic volume; LVSV = Left ventricular stroke volume; RVEF = Right ventricular ejection fraction; RVEDV = Right ventricular end diastolic volume; RVESV = Right ventricular end systolic volume; RVSV = Right ventricular stroke volume

Twenty-four patients were diagnosed as having dcSSc and 30 as having lcSSc. Digital ulcers were registered on 13 patients and 41 did not have digital ulcers. Nineteen patients had anti centromere antibodies (ACA), 18 had anti topoisomerase I antibodies (ATA), 4 had anti RNA polymerase III antibodies (ARA), 10 had anti-nuclear antibodies without ACA, ARA or ATA (ANA+) and 3 were ANA-negative (ANA-). Patients had mean disease duration (time of observation from SSc diagnosis to CMR examination) of 43 months (range 6.6–311 months).

Four patients had LGE pattern consistent with myocardial infarction (2 had subendocardial and 2 had transmural infarction) of which only one had prior suspected ischemic heart disease and 9 had fibrosis at RV insertion, leaving 37 patients without visually localized fibrosis and scar. Four patients had no gadolinium administration due to impaired kidney function (n = 1), anxiety/claustrophobia ending the examination (n = 2) or declining to receive gadolinium (n = 1).

### 3.2. CMR-FT results

#### 3.2.1. Myocardial deformation analysis in SSc patients compared to controls

Patients with SSc had lower RV longitudinal strain and strain rate compared to controls (p<0.001 and p = 0.01, respectively). All other strain and strain rate measurements were non-significant between patients and controls (Table 2).

When evaluating skin involvement, all strain and strain rate measurements were non-significant between patients with lcSSc, dcSSc or healthy controls (Table 2).

When evaluating severe microvascular involvement of the skin, all strain measurements, except LV mid circumferential strain (p = 0.02), were non-significant between patients with or without digital ulcers (Table 2).

LV longitudinal strain was lower in patients with insertion fibrosis (-18.0%) and even lower in patients with infarction (-16.6%) compared to those without fibrosis (-20.3%, p = 0.04 among the groups). LVEF, LV radial and circumferential strain as well as RV longitudinal strain did not differ among the groups. However, RVEF was lower in patients with fibrosis and infarction (Table 3).

**Table 3:** Peak systolic radial, circumferential and longitudinal left ventricular myocardial strain and strain rate and peak systolic longitudinal right ventricular free wall myocardial strain in systemic sclerosis (SSc) patients without fibrosis, with insertion fibrosis and with infarction diagnosed with late gadolinium enhancement.

	No fibrosis (n = 37*)	Insertion fibrosis (n = 9)	Infarction (n = 4)	P-value
**Left ventricle**				
Mid Radial Strain	44.2±11.4	39.6±13.4 (n = 8)	34.7±4.0 (n = 3)	0.2
Mid Circumferential Strain	-22.0±3.0	-20.7±3.8 (n = 8)	-18.7±1.2 (n = 3)	0.07
Longitudinal Strain	-20.3±3.2	-18.0±2.3	-19.6±3.3	0.04
EF	63±5	63±5	58±6	0.2
**Right Ventricle**				
Longitudinal Strain	-27.7±3.7	-26.7±4.5	-24.9±2.2	0.2
EF	61±6	53±12	55±9	0.01

Strain is measured in (%) and EF = ejection fraction expressed in mean±SD.

*4 patients had no gadolinium administration.

In disease duration correlation analysis, all correlations between disease duration and strain and strain rate measurements were non-significant (LV longitudinal strain, p = 0.8; RV longitudinal strain, p = 0.2; LV mid radial strain, p = 0.7; LV mid circumferential strain, p = 0.6; LV longitudinal strain rate, p = 0.7; LV mid radial strain rate, p = 0.3; LV mid circumferential strain rate, p = 0.5).

All strain and strain rate measurement, except LV longitudinal strain rate (p = 0.04), were non-significant when comparing groups of autoantibodies ACA, ARA, ATA, ANA+ or ANA (LV longitudinal strain, p = 0.9; LV mid radial strain, p = 0.3; LV mid circumferential strain, p = 0.08; LV mid radial strain rate, p = 0.6; LV mid circumferential strain rate, p = 0.4; RV longitudinal strain, p = 0.1).

#### 3.2.2. Variability

Intra-class correlation in inter-observer variability was <0.5 for LV longitudinal strain, RV longitudinal strain, LV radial strain and LV circumferential strain.

## 4. Discussion

In this CMR study, early cardiac involvement in cardiac asymptomatic SSc patients was documented in the RV but not in the LV. SSc patients had smaller LV/RV volumes as well as higher LVEF, but no difference in radial, circumferential or longitudinal indices, compared with age- and gender-matched healthy controls. Thirteen out of fifty SSc patients had insertion fibrosis and/or infarction, detected due to lower LV longitudinal strain, compared with those without. Finally, LV circumferential strain and LVEF were higher in SSc without, compared with those with digital ulcers.

The finding of smaller LV and RV volumes and higher LVEF could be related to heart rate. Interestingly, LV mass was not significantly different between patients and controls. SSc patients have higher heart rate than normal controls, and autonomic dysfunction with altered diastolic function and cardiac remodeling in SSc has been suggested [29]. Furthermore, vasoactive calcium channel blockers (such as the often used Nifedipine) can induce increased heart rate as a reflection of the sympathetic nerve system of vasodilatation [30]. With 50% of patients having calcium channel blockers, in our study, both SSc in itself and calcium channel blockers can cause higher heart rate. A higher heart rate decreases the diastolic filling, and this could affect the volumes and function [31]. Lastly, diffuse fibrosis could stiffen the myocardium and leads to decrease the ventricular volumes, leading to increased LVEF. In a recent study by Hromádka et al. on SSc patients [32] the conventional echocardiography parameters were similar in SSc patients and controls. However, the global longitudinal peak systolic strain (GLPS) was lower in SSc patients compared to controls and correlated negatively with native T1 (30). In our study, ventricular volumes and detection of local fibrosis were in parity with the echocardiographic results. However, in the latter study no subclinical infarction was reported.

Global longitudinal strain has an important role in identifying early myocardial dysfunction in patients with non-ischemic cardiomyopathies [33, 34]. However, in the majority of our patients left ventricular indices were not different in SSc compared to controls. This is potentially due to the selection of asymptomatic patients in which the purpose of our study was to find subclinical manifestations. Our results were in agreement with recent data showing subclinical CMR-derived myocardial deformation abnormalities in SSc patients, due to underlying fibrosis or infarction, as assessed by LGE [20], as well as parametric imaging [35]. Furthermore, LGE was significantly associated with circumferential and radial strain that is also in agreement with our results. The early detection of cardiac involvement in SSc could allow timely redirection of the management of these patients and potentially prevent the progression to cardiac damage with improved quality of life and longevity, underscoring the potential value of CMR-FT in identifying cardiac involvement in this cohort of patients.

Right ventricular deformation indices were significantly impaired in the patients’ group. SSc patients are prone to develop pulmonary hypertension and early RV involvement should always be suspected in this population. Our results are in agreement with previous echocardiography derived ones identifying early-stage impaired myocardial deformation. [36] Defining early markers of impaired RV function is crucial and may constitute a potential target for early intervention.

We aimed further to examine the potential correlation of myocardial deformation to the underlying SSc clinical status and autoimmune profile. It has been documented that cardiac manifestations occur earlier and more frequently in patients affected by dcSSc than with lcSSc [37]. Patients with lcSSc or dcSSc are characterized by clinically evident inflammatory and fibrotic processes of the skin, especially those with ARA. These mechanisms also play a major role in cardiac involvement leading to various clinical manifestations including heart failure, arrhythmias and pulmonary hypertension. Patients with dcSSc are at higher risk to develop cardiac involvement than those with lcSSc [38, 39]. However, in our study, the lack of differences between patients with lcSSc and dcSSc are consistent with a previous study in which cardiac symptoms were not found to be significantly different with the use of echocardiography derived indices between the two subtypes [40].

There were no major differences in strain among patients with different autoantibody profile. So far, there has been conflicting data in the literature whether strain is altered in patients with different phenotypes of autoantibodies [36, 39, 41]. Further large-scale research is required to offer more insight on the relative myocardial involvement in these subgroups of patients. After all, in the present study, the subgroups were rather small.

From a technical perspective, our strain measurements were expressed as the fractional change in the length (as a percentage) from the resting state (end diastole) to the state following myocardial contraction [42]. Strain analysis using this feature tracking method [43] is as feasible for the assessment of non-tagged SSFP cine CMR as for tagging CMR, indicating a unique new approach for comprehensive clinical assessment of regional cardiac function [44]. Tracking techniques have been reported more robust and reproducible [45] for global rather than regional values [46, 47].

Both echocardiography and CMR currently offer reproducible measurements of global strain values that can be applied in different clinical scenarios to assess LV and RV function [48]. CMR has an established and continuously expanding role in tissue characterization and is the modality of choice for accurate evaluation of global function using volumetric assessment. The additional value of myocardial deformation information beyond direct tissue characterization remains to be evaluated. A reliable segmental analysis is expected to render the technique clinically meaningful.

## 5. Limitations of the study

Limitations of the study were:

1. The use of multiple scanners and effects on variability of CMR measurements. From echocardiography, examining the same patient with two echo machines from different vendors can generate different strain values even when images are interpreted with the same software [49] and analysis software from different vendors can generate different strain values even from the same image data set. [50] However, we used the same software and multivendor comparison for CMR strain analysis are not available yet. Multicenter studies with multivendor assessment of LGE and myocardial at risk has been published [51] why we consider the assessment of fibrosis from three vendors as a minor limitation
2. Our study included mainly long-term SSc cardiac asymptomatic patients with a median time of 3.6 years from diagnosis. Therefore, part of the included patients had already received potentially cardiotoxic disease-modifying agents.
3. Our CMR protocol included only functional assessment as well as visual detection of focal replacement fibrosis. Parametric imaging, which is currently the most sensitive index for detection of diffuse myocardial fibrosis, irrespective of LGE, was not included in the protocol. Perfusion imaging has been the subject of previous studies by some of the authors. [17]
4. The sample size of the patient group is small but comparable to number of patients traditionally used in similar studies, given the rare nature of the entity. The control group was matched with the patient population for sex and age, although LV strain with CMR has been shown to be only sex dependent, in order to achieve the maximal accordance between the two groups.

## 6. Conclusion

A comprehensive biventricular myocardial deformation study with CMR feature tracking and LGE reveals early subclinical cardiac involvement in cardiac asymptomatic SSc with normal routine cardiac evaluation. CMR-FT contributed to differentiate the patients with silent myocardial necrosis as well as identified early RV involvement. The clinical significance of CMR deformation abnormalities for outcome and treatment goals remains to be elucidated through multicenter longitudinal studies.
